# Does Scale of Public Hospitals Affect Bargaining Power? Evidence From Japan

**DOI:** 10.15171/ijhpm.2017.29

**Published:** 2017-03-07

**Authors:** Konosuke Noto, Takao Kojo, Ichiro Innami

**Affiliations:** ^1^ Graduate School of Media and Governance, Keio University, Kanagawa, Japan.; ^2^ Center for Community Medicine, Jichi Medical University, Tochigi, Japan.; ^3^ Faculty of Policy Management, Keio University, Kanagawa, Japan.

**Keywords:** Public Hospitals, Economies of Scale, Bargaining Power, Management Responsibility, Management Efficiency

## Abstract

**Background:** Many of public hospitals in Japan have had a deficit for a long time. Japanese local governments have been encouraging public hospitals to use group purchasing of drugs to benefit from the economies of scale, and increase their bargaining power for obtaining discounts in drug purchasing, thus improving their financial situation. In this study, we empirically investigate whether or not the scale of public hospitals actually affects their bargaining power.

**Methods:** Using micro-level panel data on public hospitals, we examine the effect of the scale of public hospitals (in terms of the number of occupancy beds) on drug purchasing efficiency (DPE) (the average discount rate in purchasing drugs) as a proxy variable of the bargaining power. Additionally, we evaluate the effect of the presence or absence of management responsibility in public hospital for economic efficiency as the proxy variable of an economic incentive and its interaction with the hospital scales on the bargaining power. In the estimations, we use the fixed effects model to control the heterogeneity of each hospital in order to estimate reliable parameters.

**Results:** The scale of public hospitals does not positively correlate with bargaining power, whereas the management responsibility for economic efficiency does. Additionally, scale does not interact with management responsibility.

**Conclusion:** Giving management responsibility for economic efficiency to public hospitals is a more reliable way of gaining bargaining power in drug purchasing, rather than promoting the increase in scale of these public hospitals.

## Background

In 2013, 892 public hospitals were owned by local governments in Japan (approximately 10% of all hospitals), most of which were in a fragile financial condition. In the same year, the gross earnings of the public hospitals reached 3.955 440 trillion JPY (around 39.55 billion USD at the rate of 100 JPY to per 1 USD) and the gross costs were 3.998 363 trillion JPY (around 39.98 billion USD). The net loss was 42.92 billion JPY (around 429.2 million USD), and 52.4% of all public hospitals were reporting net losses, which were ultimately covered by taxes. This trend in the public hospital deficit has been evident for more than 30 years. The factors causing it include providing highly advanced medical care and medical services in unprofitable regions in accordance with the health policy set by both central and local Japanese governments. On the other hand, the lack of management responsibility for economic efficiency in public hospitals also has been a factor.^1^

To reverse this chronic financial deterioration, the Ministry of Internal Affairs and Communications released “The Guideline for Public Hospitals Reform” in December 2007 and “The New Guideline” in March 2015. These guidelines require local governments to formulate reform plans (specific plans to increase earnings and reduce expenses) for each public hospital. They also require evaluations and annual reports on the results. Each local government has formulated reform plans based on these guidelines.

Many of the reform plans mentioned “promoting group purchasing with other medical institutions and utilizing the gain from the economies of scale for increasing the bargaining power to obtain discounts in drug purchasing” (eg, Tomi Municipal Hospital,^2^ Kyotango City Hospital,^3^ Otsuki City National Health Insurance Hospital,^4^ Saitama Cancer Center,^5^ Nayoro City Hospitals,^6^ and many others). According to these, the intent is that if hospitals obtain such discounts in drug purchasing, their material costs would be reduced, and their financial condition would be directly improved.

The idea in these reform plans that the group purchasing (ie, increasing drug purchase volume) increases the bargaining power is based on the concept of the economies of scale. To this effect, Galbraith suggested that large buyers have an advantage in extracting price concessions from suppliers.^7^ The gain from the economies of scale in price negotiations is amounts to countervailing power, on which there already is an extensive literatures.^8-11^ These studies suggest that countervailing power does not emerge occur all circumstances but depends on other factors of the economic environment. Several empirical studies support this suggestion.^12-14^

If the economies of scale really exist in the group purchasing of drugs, the scale of hospitals should affect the bargaining power, because larger hospitals purchase drugs in larger volume, which is close to group purchasing. Although, group purchasing has been promoted in Japanese public hospitals, the relationship between the scale of public hospitals and the bargaining power has not yet been empirically analyzed. In this study, using micro-level panel data on Japanese public hospitals, we empirically investigate whether or not the scale of public hospitals affects the bargaining power in drug purchasing.

Most of the previous studies on the Japanese pharmaceutical market analyzed the effects of purchase prices on demand for drugs (prescription volumes),^15-19^ rather than how purchase prices are determined. A few theoretical studies have discussed the pharmaceutical distribution market mechanism within the Japanese drug-pricing system.^20-22^ However, these studies did not discuss the bargaining power of hospitals.

The Japan Fair Trade Commission (JFTC) conducted a questionnaire survey about drug distribution in the Japanese pharmaceutical market.^23^ The survey showed that the hospitals that adopted group purchasing accounted for 18% of all the hospitals (including private hospitals) in the year 2006. On the other hand, in the United States, 72% of all hospitals relied on GPO (Group Purchasing Organization) in the same year. Additionally, the most common answer given by medical institutions (89/274) to the question, “Why did you not adopt group purchasing” was “Even if we adopted group purchasing, we could not expect to obtain significant discounts.” Furthermore, the most common answer of wholesalers (52/79) about their price setting was “Even if the amount of the order is increases, we do not discount the selling price.” However, this questionnaire survey covers only a small portion of the total number of medical institutions and wholesalers (according to the survey in 2006, there were 9016 hospitals and 3451 pharmaceutical wholesalers in Japan) and did not conduct a detailed empirical analysis.To the best of our knowledge, this study is the first such analysis of the relationship between the scale of public hospitals and their bargaining power.

## Methods

### Data and Variables

This study uses micro-level panel data on all Japanese public hospitals, which were collected from the Yearbooks of Public Enterprises (CHIHO-KOEI-KIGYO-NENKAN) from 2004 to 2013.^24^ The yearbooks report financial and operational data of all Japanese public hospitals (including public university hospitals and independent administrative institutional hospitals) for the business years. The number of listed hospitals in 2004 was 1001, and fell to 908 in 2013. During this period, the closing or privatization of public hospitals resulted in some data loss, whereas consolidations and new establishments generated some sample data. Therefore, this panel data is unbalanced. As a result of excluding the samples with missing values and outliers (we excluded 16 samples as outliers, drug purchasing efficiency [DPE] of which exceeded 1000), the final number of hospitals for analyses is 995 and the sample size is 8338 during the estimation period (from 2004 to 2013).

In the estimations, the dependent variable should conceptually be bargaining power. Unfortunately, bargaining power associated with group purchasing is not directly observable. Therefore, we use the DPE (drug-earnings/drug-expenses × 100) of each hospital as the proxy measurement of the bargaining power. The DPE is defined by the Japanese government and reported in the yearbooks. In the Japanese pharmaceutical market, the reimbursement price of ethical drugs is officially set by the drug pricing system. On the other hand, the free market price is allowed as a procurement price (the purchasing price of hospitals from wholesalers). Therefore, the DPE indicates the average discount rate (ie, the bargaining power) gained by each hospital in the business year. When the DPE is over 100, the hospital gained discounts on average. On the other hand, when the value is below 100, the hospital purchased drugs at a higher price than the official reimbursement price.

Although the independent variable should be representing the economies of scale in the group purchasing, the data showing if any particular public hospital uses group purchasing is not available. Therefore, we use the “number of occupancy beds (NOB)” as a proxy variable for the scale of public hospitals. The “NOB” is calculated as the “number of registered beds (NRB)” × the “average bed occupancy rate (BOR)” for each hospital. The NOB can be a more realistic indicator of hospital scale, because a larger NOB means a larger volume of prescriptions and drug orders, which is nearly equal to group purchasing. If economies of scale exist in drug purchasing, the coefficient of this variable should be positive.

Additionally, we introduced a dummy variable to evaluate the effect of the presence or absence of public hospitals management responsibility for economic efficiency (economic incentive), which takes the value 1 for “Full Application public hospitals of the Local Public Enterprise Act,” and 0 for “Partial Application public hospitals.” The Partial Application is a default setting for all public hospitals, where no one takes the responsibility for inefficient management, and losses are eventually covered by taxes. On the other hand, the Full Application public hospitals are required to appoint a manager with management responsibility and discretionary power over budget and personnel matters. Therefore, the Full Application public hospitals have an institutional framework (economic incentive) for efficient management compared to the Partial Application ones. Hence, we assume that the Full Application hospitals try to obtain discounts in drug purchasing and expect the coefficient of this dummy variable to be positive. The number of Full Application hospitals has gradually increased from around the year 2000 in response to the previously mentioned financial deterioration. However, it was still only around 30% of all public hospitals in 2013.

Furthermore, we also introduced the interaction term between the NOB and the Full Application Dummy to the model. The coefficient of the interaction term shows the effect of scale on the DPE in Full Application hospitals. Moreover, we added “Year Dummies” and “Region (Prefecture) Dummies” to the models in order to control for such effects as the drug price revision every two years and the competitive environment in the certain regions which has been pointed out in previous studies.^13^

Table 1 reports the descriptive statistics of the above variables. The DPE and the NOB are converted into the logarithmic form in the following estimations, and which also are listed in parentheses. The average value of DPE is 108.75 (8.75%), which is relatively close to the overall average value of the DPE (including private hospitals and pharmacies) in the periods.^25^ Moreover, the Full Application hospitals constitute 34% of the total samples.

**Table 1:** Descriptive Statistics

**Variables**	**Observation**	**Mean**	**SD**	**Min**	**Max**
DPE (ln)	8338	108.75 (4.65)	33.75 (0.27)	4.50 (1.50)	988.10 (6.90)
NRB	8338	239.81	188.76	20	1368
Average BOR	8338	0.73	0.16	0.09	1.04
NOB (NRB × BOR) (ln)	8338	182.41 (4.80)	158.82 (0.96)	0.34 (-1.07)	977.96 (6.89)
Full Application Dummy	8338	0.34	0.47	0	1

Abbreviations: DPE, drug purchasing efficiency; NRB, number of registered beds; BOR, bed occupancy rate; NOB, number of occupancy beds; SD, standard deviation.

In advance of the following estimations, we confirm the stationarity of ln(DPE) and ln(NOB), using Fisher type-augmented Dickey-Fuller (F-ADF) test to avoid the spurious correlation.^26^ The test results clearly reject the null hypothesis that these variables contain a unit root (for both variables, *P* < .00). Therefore, we concluded that the ln(DPE) and the ln(NOB) are stationary throughout the period*.*

### Model

Using these variables, we conducted the estimations by pooling ordinary least squares (OLS), fixed effects model, and random effects model. First, the pooling OLS is as formula (1), which is to estimate the coefficient by pooling all of the panel data and ignores the heterogeneity of each hospital.

(1) $${\text{ln D}}_{it}\text{=}\alpha \text{+}\beta_{1}{\text{lnB}}_{it}\text{+}\beta_{2}F_{it}\text{+}\beta_{3}{\text{(lnB}}_{it}\times {\text{F}}_{it}{\text{)+T}}_{t}{\text{+R}}_{i}{\text{+u}}_{it}$$

where the subscript *i* = 1,…,995 corresponds to individual hospitals; and *t* = 2004 ,…, 2013 corresponds to each year; D_it_ is the DPE; B_it_ is the NOB; F_it_ is the Full Application Dummy; T_t_ are a set of year dummies; R_i_ are a set of region dummies; α is the constant term; and u_it_ is the error term. Since the variables were converted into the logarithmic form, each coefficient β is interpreted as elasticity.

Next, the fixed effects model and the random effects model are in formula (2), which takes into account the unobserved heterogeneity of each hospital.

(2) $$\begin{array}{l} {\text{ln D}}_{it}\text{=}\alpha \text{+}\beta_{1}{\text{lnB}}_{it}\text{+}\beta_{2}F_{it}\text{+}\beta_{3}{\text{(lnB}}_{it}\times {\text{F}}_{it}{\text{)+T}}_{t}{\text{+R}}_{i}\text{+}\varepsilon_{it}\\ \varepsilon_{it}=\mu_{i}+v_{it} \end{array}$$

Where ε_it_ is error term, and µ_i_ is the unobserved time-invariant individual effects. In the fixed effects model, the unobserved time-invariant individual effects, µ_i_ is assumed to be correlated with independent variable, and this model is estimated by the first-difference (FD) estimation. Lastly, random effects model also takes into account the unobserved time-invariant individual effects µ_i_. However, the model assumes that µ_i_ is dependent from the independent variable and this model is estimated by the generalized least squares (GLS).

Additionally, we conducted model selection tests among the above three models. First, the F test and the Breusch-Pagan (B-P) test are used to compare the pooling OLS with the fixed effects model and the pooling OLS with the random effects model respectively. Next, using the Hausman specification test, the fixed effects model and the random effects model are compared. The tests determine the relative suitability of the models. For more details about the panel data analysis and model selection tests, references are available in the texts.^27,28^

### Controlling for Heterogeneity by Fixed Effects Model

Generally, one of the most important conditions for estimating hospital efficiency is to ensure the homogeneity of hospitals in the sample data.^29^ However, Japanese public hospitals exhibit significant heterogeneity in their facilities characteristics and roles. They also vary in the scale of hospital beds (from 20 to 1368 in the data set) and provide different services in terms of the health policy (eg, some public hospitals provide highly advanced medical care, while others play a major role in community-based health care or provide medical services in unprofitable regions). Such heterogeneity may cause an estimation bias, which can do harm to the validity of the estimation results. However, fixed effects model can control this bias as the unobserved time-invariant individual effects µ_i_ of each hospitals by estimating the first differences models and eliminating the µ_i_ from the model. Therefore, we can estimate more reliable parameters in an insufficiently homogenized situation.^30^

## Results

Table 2 shows these estimation results. First, the results of the model selection tests (F test, B-P test and Hausman specification test) show that the fixed effects model is the most suitable model for the data. The results indicate that the individual effects of each hospital on DPE are observed throughout the period. Hence, we are focusing on the results of the fixed effects models (2), (5), and (8). Next, the coefficients of the NOB are not significant in model (2), and (5). However, in the model (8) the coefficient is significantly negative. The results suggest that the scale of public hospitals and the DPE have no positive correlation; rather it has a negative correlation in the more controlled model (8). The coefficient parameters show that when the NOB increases by 1%, the DPE decreases by only 0.11%.

**Table 2:** Estimation Results

**Dependent Variable: ln(DPE)**							
**Variables**	**OLS (1)**	**FE (2)**	**RE (3)**	**OLS (4)**	**FE (5)**	**RE (6)**	**OLS (7)**	**FE (8)**	**RE (9)**
ln(NOB)	-0.07^***^	0.01	-0.04^***^	-0.06^***^	-0.02	-0.03^***^	-0.08^***^	-0.11^***^	-0.07^***^
Full App Dummy (F)				0.16^***^	0.37^***^	0.33^***^	0.11^***^	0.49^***^	0.38^***^
ln(NOB) × (F)				-0.03^***^	-0.09^***^	-0.08^***^	-0.02^**^	-0.09^***^	-0.07^***^
Year Dummies (9)	No	No	No	No	No	No	Yes	Yes	Yes
Region Dummies (46)	No	No	No	No	No	No	Yes	No	Yes
Observation	8338	8338	8338	8338	8338	8338	8338	8338	8338
Number of Hospitals	995	995	995	995	995	995	995	995	995
Adjusted R^2^	0.06			0.06			0.15		
R^2^（within）		0.01	0.01		0.03	0.03		0.14	0.13
R^2^（between）		0.08	0.08		0.02	0.04		0.07	0.12
R^2^（overall）		0.06	0.06		0.03	0.04		0.09	0.14
F test	0.00	0.00	0.00
Hausman test	0.00	0.00	0.00						
B-P test	0.00	0.00	0.00						

Abbreviations: OLS, ordinary least squares; DPE, drug purchasing efficiency; FE, fixed effects; RE, random effects; B-P, Breusch-Pagan. Note: *,**,*** denote significance at 10%, 5%, and 1% levels, respectively. The constant terms are omitted. All estimations and tests were performed by Stata/SE 14.0.

On the other hand, the coefficients of the “Full Application Dummy of the Local Public Enterprise Act” show significantly positive correlation in all models as expected. The differences in the DPE between of the Full Application hospitals and the Partial Application hospitals are approximately 37% and 49% in models (5), and (8), respectively.

The coefficients of the interaction term between NOB and Full Application Dummy are slightly negative at the 1% significant level in all cases. This result shows that the scale of public hospitals and the DPE have also no positive interaction in the Full Application hospitals.

## Discussion

The NOB coefficients indicate that the scale of public hospitals does not positively affect the bargaining power in purchasing drugs, which means that the scale of public hospitals does not matter in drug purchasing negotiations. One possible reason for this is that they did not negotiate with wholesalers in the first place for lack of the economic incentive (management responsibility for efficient management).^1^

On the other hand, the coefficients of “Full Application Dummy variable of the Local Public Enterprise Act,” which represent the management responsibility of public hospitals show a significantly positive value in all models. The results indicate that public hospitals with management responsibility did negotiate a purchase price and could obtain substantial discounts relative to public hospitals with no management responsibility. In other words, Full Application of the Local Public Enterprise Act seems to play a role as an incentive to make public hospitals put more effort into price negotiations.

Furthermore, the small negative coefficients of the interaction term between NOB and Full Application Dummy suggest that the scale of Full Application public hospitals has no positive effect on their bargaining power as well. In other words, it is not economies of scale but the economic incentive (management responsibility for efficient management) that enables to obtain significant discounts.

The open question is why the findings indicate that public hospitals do not exploit gains from economies of scale in price negotiations, to which there are three possible answers. One is that they simply do not utilize the economies of scale because they do not have the economic incentive to use this gain.

The second one is the omitted-variable bias. The low level coefficients of determination (R^2^= 0.06~0.14) suggest that there may be other factors affecting the DPE. Usually, the unobserved individual effect in fixed effects model improves this bias. However, the previous empirical study suggested that supplier competition (interaction with the substitution opportunities for buyers) is a prerequisite for the economies of scale in drug purchasing.^13^ In this study, the competitive environment in the region (Prefecture) has been roughly controlled by the region dummy variable or the unobserved individual effect. Nevertheless, we were unable to investigate the interaction between a hospital’s scale and their substitution opportunities due to data constraints. Hence, a more detailed analysis is required for this point.

The third reason is that large scale hospitals provide highly advanced medical care which obliges them to use new drugs in patent time (no competition with other drugs) than generic drugs, which in turn leads to lower discount rate. The negative correlations between the scale of public hospitals and their bargaining power in the result indicate this possibility.

## Conclusion

In this study, using micro-level panel data on all Japanese public hospitals, we empirically investigated whether or not the scale of public hospitals affects their bargaining power. Additionally, we evaluated the effects of the presence or absence of management responsibility for economic efficiency in public hospitals and its interaction with the scale effects on the bargaining power. The results indicate no positive correlations between scale and bargaining power, whereas there are correlations between management responsibility and bargaining power. Simply put, the scale does not matter, but economic incentive does. This suggests that giving management responsibility to public hospitals is a more reliable way of increasing bargaining power in drug purchasing, thus improving their financial situation, rather than promoting the increase in the scale of public hospitals.

Therefore, increasing the number of Full Application hospitals of the Local Public Enterprise Act (currently, as indicated, it remains around 30% as indicated) appears to improve the overall financial condition of public hospitals. Since the financial improvement of public hospitals has been an urgent issue in Japan, this study provides an empirical evidence to support the public hospital reform plans.

However, the study has some limitations as follows. First, the low coefficients of determination in the estimation results indicate that there are other factors affecting the DPE, as previously mentioned. Therefore, we should scrutinize other factors associated with the bargaining power of public hospitals and introduce a new variable to illustrate the determinants. Second, we could not directly examine the relationships between the bargaining power of public hospitals and the group purchasing because of data limitation. Hence, there is scope for future research on drug purchasing with more detailed data.

## Acknowledgements

This paper was partially supported by Keio Research Institute at SFC, Academic Exchange Grants 2015, 2016 (Grant Project: Health Policy and Management) and the Japan Society for the Promotion of Science, Grants-in-Aid for Scientific Research (KAKENHI), Grant Number 14J10648.

## Ethical issues

There is no ethical issues. All data in this paper is publicly available.

## Competing interests

Authors declare that they have no competing interests.

## Authors’ contributions

KN conducted the research design and writing. The dataset was constructed by KN and TK. The estimation models was developed by KN, TK, and II. All statistical estimations and tests were conducted by KN. The conclusion and implications were discussed by KN, TK, and II.

## Authors’ affiliations

^1^Graduate School of Media and Governance, Keio University, Kanagawa, Japan. ^2^Center for Community Medicine, Jichi Medical University, Tochigi, Japan. ^3^Faculty of Policy Management, Keio University, Kanagawa, Japan.

## Key messages

**Implications for policy makers**

- The scale of public hospitals does not positively correlate with bargaining power, whereas management responsibility for economic efficiency does.
- Giving management responsibility for economic efficiency to public hospitals by increasing the Full Application hospitals of the Local Public Enterprise Act may be a more reliable way of improving the financial performance than promoting an increase in the scale of public hospitals.

**Implications for public**

The financial improvement of public hospitals has been an urgent issue in Japan. This study provides empirical evidence that supports the government’s public hospital reform plans.

## References

[1] Hori M (2007). The role of public hospital in Japanese health care providing system. Government Auditing Review.

[2] Tomi City Office. The Reform Plan of Tomi Municipal Hospital. http://hospital.city.tomi.nagano.jp/simin/kaikakuplan-gaiyo.pdf. Accessed May 1, 2016. Published 2009.

[3] Kyotango City Office. The Reform Plan of Kyotango City Riyasaka Hospital. http://www.city.kyotango.lg.jp/cms/shisei/singikai/kekka/iryokaikakukaizen/documents/1304.pdf. Accessed May 1, 2016. Published 2008.

[4] Otsuki City Office. The Reform Plan of Otsuki City National Health Insurance Hospital. http://www.town.taiki.hokkaido.jp/soshiki/byouin/sonota.data/kaikakuplan.pdf. Accessed May 1, 2016. Published 2009.

[5] Local Government of Saitama Prefecture. The Reform Plan of Saitama Cancer Center. http://www.soumu.go.jp/main_sosiki/c-zaisei/hospital/pdf/saitama_h21/saitama090525_02.pdf. Accessed May 1, 2016. Published 2009.

[6] Nayoro City Office. New Reform Plan of Nayoro City Hospitals Management. https://www.nayoroch.jp/hotnews/files/00000300/00000366/kaikaku2016-6-2.pdf. Accessed May 1, 2016. Published 2016.

[7] Galbraith JK. American Capitalism: The Concept of Countervailing Power. New Brunswick: Transaction Publishers; 1993.

[8] Snyder CM (1996). A Dynamic Theory of Countervailing Power. Rand J Econ.

[9] Snyder CM (1998). Why do larger buyers pay lower prices? Intense supplier competition. Econ Lett.

[10] Adilov N, Alexander PJ (2006). Horizontal merger: Pivotal buyers and bargaining power. Econ Lett.

[11] Inderst R, Wey C (2007). Buyer power and supplier incentives. Eur Econ Rev.

[12] Hans-Theo N, Bradley JR, Christopher MS. Do Buyer-Size Discounts Depend on the Curvature of the Surplus Function? Experimental Tests of Bargaining Models. EconWPA; 2003.

[13] Ellison SF, Snyder CM (2010). Countervailing power in wholesale pharmaceuticals. J Ind Econ.

[14] Ruffle BJ (2013). When do large buyers pay less? Experimental evidence. J Ind Econ.

[15] Anegawa T (1999). Empirical study of the demand and price of pharmaceutic als: a case of pharmaceuticals for cardiovascular system. Iryo To Shakai.

[16] Anegawa T (2002). Price regulation and demand for pharmaceuticals reconsidered: a case of pharmaceutical fsor cardiovascular System. Iryo To Shakai.

[17] Onda M, Sato M (2002). Official Price Tariff and Demand of Pharmaceuticals. Japanese Journal of Health Economic and Policy.

[18] Seiritsu O, Takehiko H (
Inc
2003
). Why Do the Japanese Spend So
Much on Drugs?. National Bureau of Economic Research,.

[19] Iizuka T (2009). Generic entry in a regulated pharmaceutical market. Japanese Economic Review.

[20] Inoue T, Tezuka K (1998). An analysis of the medical supplies distribution system in Japan. Journal of Business and Management.

[21] Inoue T, Tezuka K (2002). An empirical study of the medical supplies distribution system in Japan. Japanese Journal of Health Economic and Policy.

[22] Tanno T, Hayashi Y (2014). Bargaining power and NHI drug price standard in ethical pharmaceutical distribution. Stud Appl Econ.

[23] Japan Fair Trade Commission. Report on the Distribution of Prescription Drugs (Iryoyouiyakuhin no ryutsujittaini kansuru chousahoukokusho). http://www.jftc.go.jp/houdou/pressrelease/cyosa/cyosa-ryutsu/h18/06092702.files/06092702-hontai.pdf. Accessed May 1, 2016. Published 2006.

[24] Ministry of Internal Affairs and Communications. Yearbook of Local Public Enterprise. http://www.soumu.go.jp/main_sosiki/c-zaisei/kouei_kessan.html. Accessed May 1, 2016.

[25] Ministry of Health, Labour and Welfare. Time Series of Average Drug Purchasing Efficiency (KAIRIRITSU). http://www.mhlw.go.jp/file/05-Shingikai-12404000-Hokenkyoku-Iryouka/0000057384.pdf. Accessed May 1, 2016.

[26] Choi I (2001). Unit root tests for panel data. J Int Money Finance.

[27] Baltagi BH. Econometric Analysis of Panel Data. 5th ed. Wiley; 2010.

[28] Wooldridge JM. Econometric Analysis of Cross Section and Panel Data. 2nd ed. MIT Press; 2010.

[29] Newhouse JP (1994). Frontier estimation: How useful a tool for health economics?. J Health Econ.

[30] Kawaguchi H. Study on Estimation of the Efficiency of Municipal Hospitals Using Panel Data (Panerudata womochiita jichitai byōin no kōritsusei no suitei ni kansuru kenkyū). Health efficiency measurement :the methods and problems (iryō no kōritsuseisokutei - sono shuhō to mondaiten). Published 2008.

